# Metastatic Renal Cell Cancer With Pancreatic Mass

**DOI:** 10.7759/cureus.27119

**Published:** 2022-07-21

**Authors:** Sushant Chaudhary, Subhash Chander, Winston Magno, Praneet Wander

**Affiliations:** 1 Internal Medicine, Saint Mary's Hospital, Waterbury, USA; 2 Pathology, Saint Mary's Hospital, Waterbury, USA; 3 Gastroenterology and Hepatology, Saint Mary's Hospital, Waterbury, USA

**Keywords:** pancreatic duct dilation, secondary pancreatic neoplasm, pancreatic mass, renal cell cancer metastasis, metastatic pancreatic mass

## Abstract

A pancreatic mass is mostly discovered late in the course of the disease and is usually asymptomatic in the early stages. In rare cases, a pancreatic mass may be metastatic, and presentation may depend on the presence and locations of other metastasis or to the primary lesion. Renal cell cancer is the most common tumor presenting as metastatic pancreatic mass. Most metastases occur within the first ten years after diagnosis. We present a case of metastatic renal cell cancer to the contralateral adrenal and pancreas causing pancreatic duct dilation, 15 years after radical nephrectomy.

## Introduction

The pancreas is an uncommon site of metastatic disease. Metastasis in the pancreas is seen only in 2-4% of patients with malignant lesions of the pancreas [1]. Metastasis may be isolated to the pancreas or may have spread to other organs at the time of diagnosis. Of the known neoplasms, renal cell cancer (RCC) most commonly metastasizes to the pancreas [1]. Almost 63% of metastasis to the pancreas arises from RCC [2,3]. Metastasis can be synchronous but metachronous lesions have been reported more often up to 10 years after radical nephrectomy [4]. We report a case of metastatic renal cell cancer to the pancreas 15 years after radical nephrectomy.

## Case presentation

**Figure 1:**
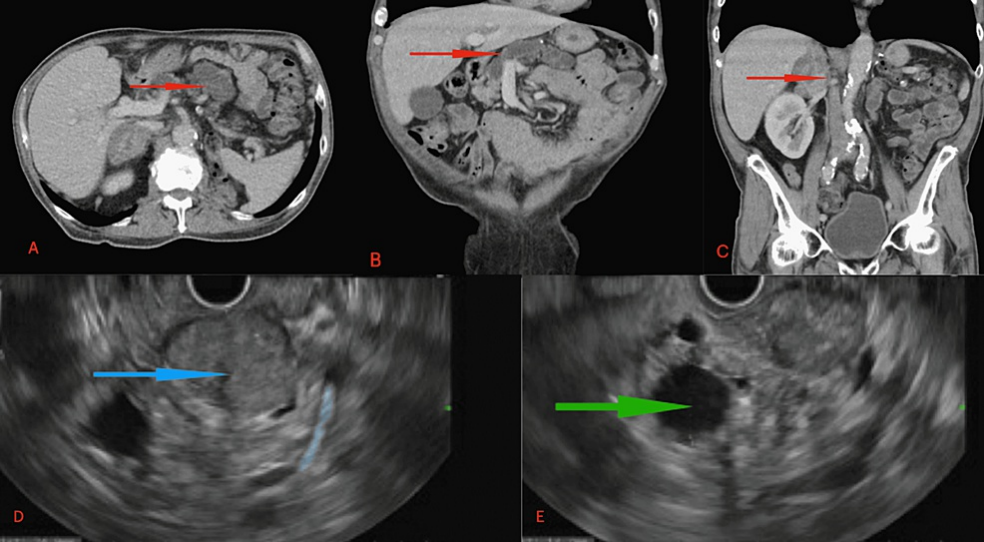
CT (panels A,B,C) and EUS (panels D,E) findings. Image A shows the mass in the pancreatic head (arrow with red head) with a dilated pancreatic duct seen in image B (arrow with red head). Adrenal metastasis is seen in image C (red arrow). On EUS, the mass appears well circumscribed in image D (blue arrow) with dilated pancreatic duct seen (green arrow) in the head of pancreas in image E EUS: endoscopic ultrasound (EUS)

**Figure 2:**
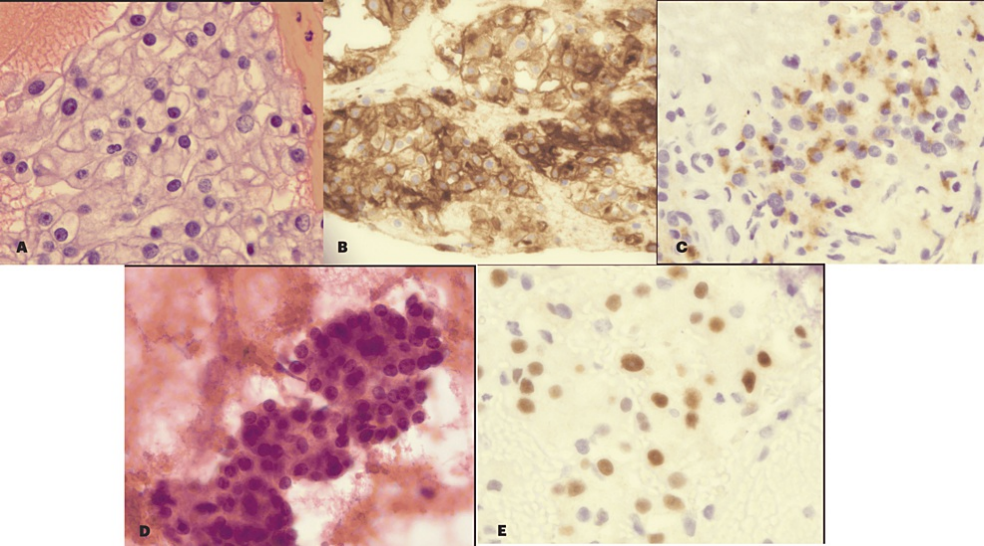
Histopathology images (panels A,B,C) and cytology (panels D). (A) H&E preparation of cell bloc shows severe anisocytosis with clear cytoplasm, large nuclei, and prominent nucleoli. Also seen are (B) CD10+ cells, (C) renal cell cancer antigen + cells, and (E) PAX8+ cells. (D) FNA of the tissue shows cells with prominent nucleoli H&E: hematoxylin and eosin; FNA: fine needle aspiration All images are with 400X magnification

A 61-year-old man with a past medical history of RCC, status post left radical nephrectomy 15 years ago, presented to the Emergency Department with a dull pain in right lower abdominal for one day. He did not receive any adjuvant therapy following the surgery. The patient was followed by his urologist for nine years after his nephrectomy without any evidence of metastasis. He denied any other abdominal symptoms or weight loss. He continued to smoke intermittently up to half a pack of cigarettes daily. His examination was unremarkable but he was noted to have lipase of 213U/L (10-160 U/L) and bilirubin of 1.1 mg% (0.1-1 mg%). He underwent a CT scan of his abdomen and was noted to have bulky, enhancing, soft tissue mass (~6.7 cm), with dilated pancreatic duct of 1.5 cm (Figure 1). He was also noted to have a right adrenal mass, with the right kidney within normal limits (Figure 1). The patient then underwent endoscopic ultrasound. The lesion appeared well circumscribed and previously known pancreatic duct dilation was noted. Fine needle biopsy of the mass was done, which showed numerous atypical cells with clear cytoplasm, round irregular nuclei with prominent nucleoli, suggestive of clear cell renal cell carcinoma (Figure 2). On immunohistochemistry, the tumor was positive for CD10, renal cell carcinoma antigen, and PAX 8 (Figure 2)

## Discussion

Synchronous metastasis to the pancreas and contralateral adrenal gland has been described [5]. However, metachronous RCC with pancreatic and adrenal metastasis, 15 years after radial nephrectomy is rare. About 230 cases of pancreatic metastasis occurring up to 10 years after nephrectomy have been reported and most lesions were multiple without any predilection for a specific part of pancreas [6]. Our patient had single pancreatic metastasis with adrenal involvement. The histopathology showed clear cell variant of RCC, the same as the original pathology of the resected renal mass. This is in agreement with the literature reviewed, as the most common variant of the RCC to metastasize [5,6]

Our patient presented with vague abdominal symptoms but was noted to have elevated lipase and dilation of main pancreatic duct. Due to a remote history of nephrectomy and the presenting complaints, pancreatic primary was suspected. However, on endoscopic ultrasound the patient was noted to have a well circumscribed lesion in the head of pancreas with dilated pancreatic duct (Figure 1). Regular borders and absence of pancreatic duct dilation suggested metastatic disease. Our patient had well circumscribed lesion but with dilated duct and atrophic distal pancreas.

Most of the pancreatic metastasis occur remotely after the primary renal cancer has been addressed. It has been reported to occur 10 years after nephrectomy but our patient had metastasis 15 years later without any loco-regional recurrence [5,6]. We believe that the spread is hematogenous with cancer cells lying dormant due to unclear reasons. Although ipsilateral adrenal metastasis is more likely in patients with large RCC, upper pole tumors, and left side lesions, contralateral adrenal metastasis also have been reported [7]. Besides anatomical reasons, the affinity of RCC cells for adrenal gland has also been postulated [7]. Proximity of the left adrenal gland may have a role to play in spread of cancer to the body and tail of pancreas but the spread of the RCC to the head of pancreas cannot be explained by causes other than hematogenous spread. Like adrenals, pancreas may also have high affinity for RCC since almost 63% of the pancreatic metastasis are renal in origin. CT and MRI remain crucial in diagnosing pancreatic masses. Endoscopic ultrasound is extremely important and allows to evaluate the extent of the disease, involvement of portal vein/superior mesenteric artery, and presence of enlarged lymph nodes, all of which are a must for evaluating the surgical candidature [8,9]. This is in addition to providing tissue diagnosis which helped us establish the diagnosis of metastasis.

## Conclusions

In patients presenting with pancreatic mass, even with a remote history of RCC, endoscopic ultrasound and biopsy of the mass should be considered to evaluate the lesions since metastatic disease is very common (especially with the clear cell variant of RCC). Well circumscribed lesions are more likely to be metastatic in nature. Dilated pancreatic duct does not rule out metastatic disease. Patients with RCC can present even after 15 years of being disease free and in absence of locoregional recurrence.
